# Oligomeric proanthocyanidins (OPCs) target cancer stem-like cells and suppress tumor organoid formation in colorectal cancer

**DOI:** 10.1038/s41598-018-21478-8

**Published:** 2018-02-20

**Authors:** Shusuke Toden, Preethi Ravindranathan, Jinghua Gu, Jacob Cardenas, Madelaine Yuchang, Ajay Goel

**Affiliations:** 1 Center for Gastrointestinal Research, Translational Genomics and Oncology, Baylor Scott & White Research Institute and Charles A Sammons Cancer Center, Baylor University Medical Center, Dallas, TX 75246 USA; 2 Baylor Scott & White Research Institute, Dallas, Texas USA

## Abstract

Proanthocyanidins are a heterogeneous group of flavan-3-ol or flavan-3,4-diol oligomers present in various fruits and vegetables. In particular, the smaller oligomeric subset of proanthocyanidins, termed the oligomeric proanthocyanidins (OPCs) appear to have potent anti-tumorigenic properties, but the underlying mechanisms for their effectiveness remain unclear. Herein, we utilized a series of *in vitro, in viv*o and patient-derived organoid approaches to systematically investigate the chemoprotective role of OPCs in colorectal cancer. OPCs exerted anti-tumorigenic effects through inhibition of cellular proliferation, and induced apoptosis and cell cycle arrest. Intriguingly, OPCs suppressed spheroid derived cancer stem-like cell formation and decreased the expression of intestinal cancer stem cell markers including LGR5, CD44 and CD133. Mechanistically, RNA-sequencing results confirmed that OPCs prominently interfered with developmental and self-renewal pathways and identified several self-renewal associated oncogenes targeted by OPCs. Furthermore, OPCs inhibited Hippo pathway through downregulation of its key transcriptional regulators, YAP and TAZ. Finally, we confirmed anti-tumorigenic effects of OPCs using multiple xenograft experiments and recapitulated its protective effects using patient-derived colorectal tumor organoids. Collectively, we have comprehensively assessed anti-tumorigenic properties of OPCs and our data throws light on previously unrecognized chemopreventive mechanisms of OPCs highlighting its therapeutic potential.

## Introduction

**Figure 1:**
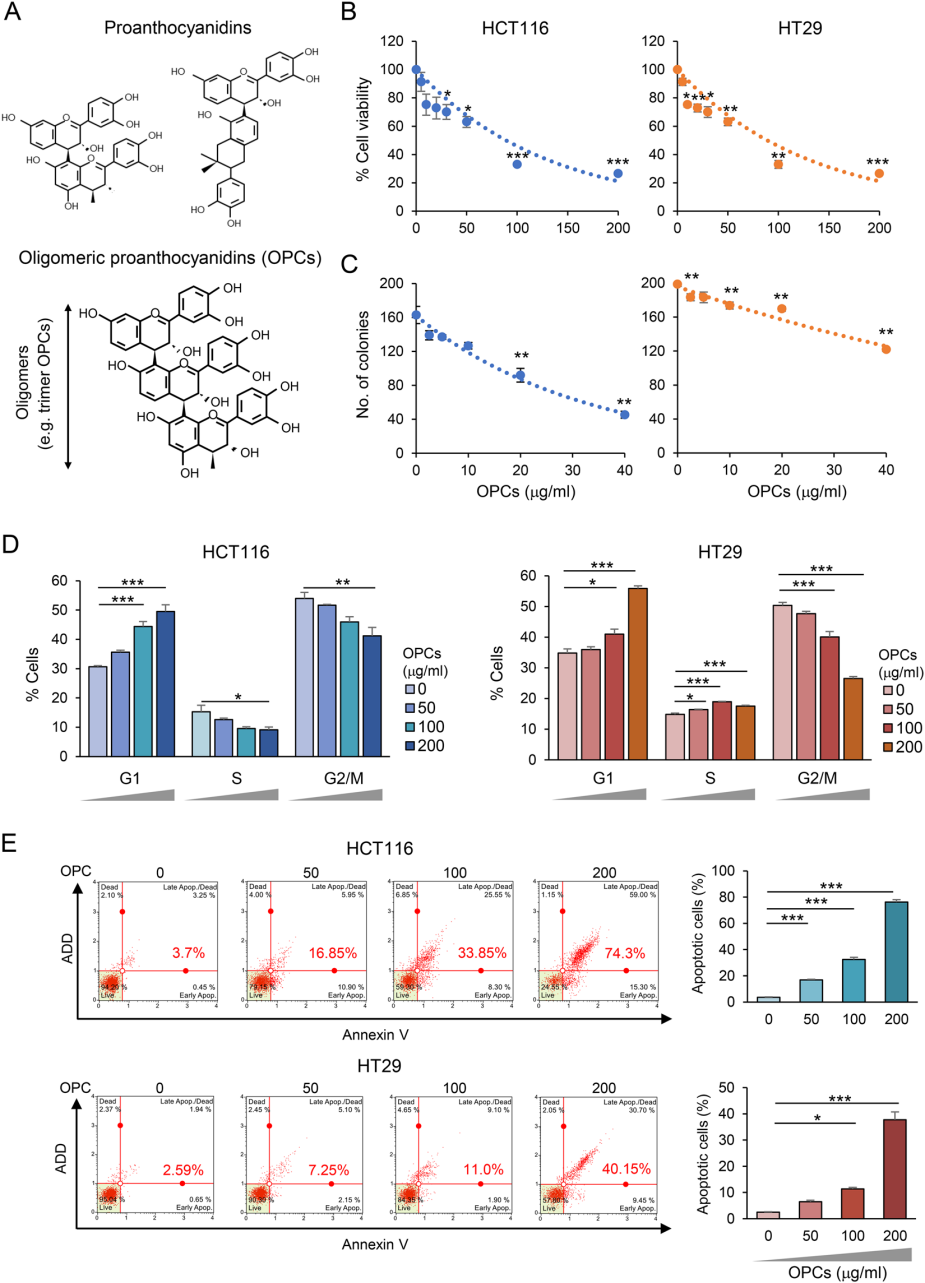
OPCs exert anti-tumorigenic properties in CRC cell lines. (**A**) Structural representation of basic monomeric units of proanthocyanidins (top) and an example of OPCs (bottom). (**B**) Cell viability, (**C**) colony formation, and (**D**) cell cycle analysis of HCT116 and HT29 cells treated with 0–200 µg/ml of OPCs. (**E**) Left: Representative apoptosis profile for cells treated with 0–200 µg/ml OPCs. ADD and Annexin V plotted for each cell along the y and the x axes respectively. Right: Quantitated apoptotic cells with different doses of OPCs. *p < 0.05, **p < 0.01 ***p < 0.001 compared to control treatments.

Colorectal cancer (CRC) is the second leading cause of cancer-related mortality in the United States^1^; however, most sporadic CRCs are potentially preventable through lifestyle and dietary modifications. In the context of cancer prevention, various botanical compounds are being extensively studied and clinically evaluated for their anti-tumorigenic properties^2–4^. One such group of cancer preventative compounds that is present naturally in fruits and vegetables such as cranberries, grape seeds and beans, are ‘proanthocyanidins’^5,6^. Chemically, these proanthocyandins are polymers of flavan-3-ol and/or flavan-3,4-diol molecules. While larger proanthocyanidins polymers are difficult to be absorbed by the body, shorter oligomers (dimers, trimers and tetramers), often referred to as ‘oligomeric proanthocyanidins (OPCs)’, have superior solubility and bioavailability owing to their smaller size (Fig. 1A). Anti-tumorigenic properties of proanthocyanidins as a broad family of polyphenols is well recognized^7–9^, however the chemopreventive efficacy of OPCs is attractive, albeit remains to be assessed comprehensively.

A major contributor to tumor initiation, metastasis and acquired resistance to chemotherapeutic drugs is believed to be orchestrated by a small subset of cancer cells, termed “cancer stem cells”^10,11^. Although the definition of cancer stem cells remains ambiguous, targeting of cancer stem cells has become one of the most popular cancer therapeutic strategies^12^. The discovery of major cancer stem cell regulatory pathways such as Wnt, Notch and Hedgehog has clarified to some degree the underlying mechanisms by which some cancer cells acquire stem cell-like features^13^. However, subsequent studies revealed that cancer stem cells are regulated by a more dynamic and complex network than previously thought, and are influenced by various genetic, epigenetic and environmental factors^14^. Despite these complexities, dietary agents such as curcumin, green tea extract and grape seed extract have emerged as promising strategies for targeting cancer stem cells^15–19^. While the efficacy of these botanicals is relatively moderate compared to chemotherapeutic drugs, their low toxicity, cost-effectiveness and other health benefits make them attractive supplements for both cancer prevention and treatment. Further understanding of their molecular mechanisms could provide additional insights into development of more potent synthetic therapeutic drugs that specifically target cancer stem cells and spare the non-cancerous cells.

Over the last few years, tumor organoid models have emerged as fascinating tools for linking basic biology to clinical applications. These sophisticated model allows maintenance and expansion of stem cells in a 3D culture, providing a physiologically superior model compared to the conventional monolayer cell culture platforms. Furthermore, organoid models allow a more precise investigation of tissue renewal in response to various drug treatments, analogous to *in vivo* mouse models; hence providing an important bridge between traditional 2D cultures and pre-clinical animal models^20^. Accordingly, tumor organoid models are becoming accepted as a facile model to study various diseases, in particular, human cancers^21^. Considering some of the skepticism surrounding the use of botanicals as chemopreventive agents and the lack of well-controlled clinical trials, utilization of patient-derived tumor organoids provides a unique avenue for investigating the chemopreventive efficacy of natural compounds. However, even though such patient-derived organoids have gradually been used for pharmaceutical drug screening purposes, these have not been utilized to assess the chemopreventive efficacy of natural compounds. In the current study, we initially assessed the anti-tumorigenic properties of OPCs in CRC using a series of *in vitro* models, followed by validation of our findings in an animal model, which was finally once again validated in tumor organoids derived from CRC patients. Using these approaches, we established the *modus operandi* of OPCs as induction of apoptosis and modulation of cell cycle arrest, and demonstrated that OPCs inhibit the formation and proliferation of cancer stem cells. Furthermore, gene-expression profiling using RNA-sequencing revealed that OPCs predominantly suppressed developmental and self-renewal pathways including inhibition of Hippo signaling pathway. Collectively, we have comprehensively demonstrated the anti-tumorigenic properties of OPCs using multiple models, including patient-derived tumor organoids, highlighting the potential clinical usefulness of OPCs as chemopreventive agents in colorectal cancer.

## Results

### OPCs exert anti-tumorigenic effects in colorectal cancer cells

Anti-tumorigenic properties of proanthocyanidins is becoming an active area of research investigations in various cancers^5–9,22,23^. In the present study, we aimed to evaluate the anti-cancer properties of a subset of proanthocyanidins, OPCs, in CRC. To ensure representation of both, microsatellite stable (MSS) and microsatellite unstable (MSI) types of CRCs in our study, we performed our assays in HT29 (MSS) and HCT116 (MSI) cell lines. Furthermore, we purposely chose the doses of OPCs to be consistent with previous studies^9,22,23^. First we investigated whether OPCs inhibit cellular proliferation of CRC cells. MTT assays revealed that OPCs significantly inhibited cellular growth of both cell lines dose-dependently (Fig. 1B). Consistent with the results of the proliferation assays, clonogenicity of CRC cells was inhibited, with significant growth suppression observed for 20 and 40 µg/ml OPCs treatment groups in both cell lines (all p < 0.01; Fig. 1C). Cell cycle analysis revealed that OPCs induced G0/G1 arrest in both cell lines in 100 µg/ml OPC treatment groups (All p < 0.05) further confirming the growth inhibitory properties of OPCs (Fig. 1D). Furthermore, the increase in the fraction of cells that underwent apoptosis upon OPC extract treatment as measured by Annexin V-based flow cytometric assay, corresponded with the OPC-induced cytotoxicity in both cell lines (Fig. 1E). Collectively, these data indicate that OPCs exert their anti-tumorigenic effects in CRC cells by modulating cell cycle dynamics and inducing apoptosis.

### OPCs inhibit formation of cancer stem cells

**Figure 2:**
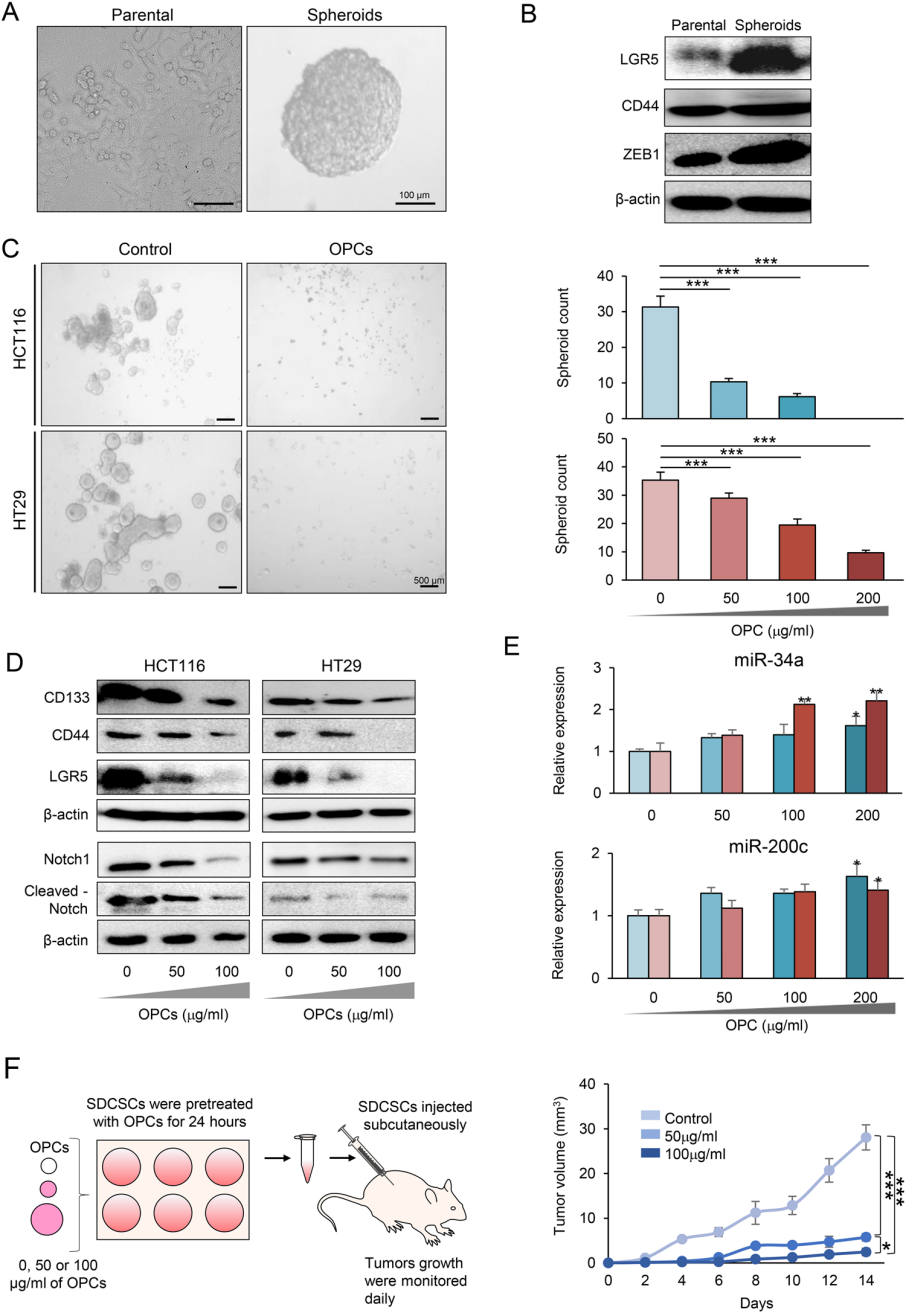
OPCs inhibit cancer stem-like cell formation. (**A**) Images of differentiated and adherent parental cells (left) and spheroids derived from parental HCT116 cells (right). (**B**) Protein expression of stem-cell markers LGR5, CD44 and ZEB1 in parental HCT116 cells and spheroids (The full-length western blots images are shown in supplementary Fig. S2). (**C**) Left: Representative images of spheroids treated with 200 µg/ml of OPCs. Right: Quantification of HCT116- and HT29-derived spheroids with OPCs treatment (right). Indicated dose in µg/ml. (**D**) Protein expression of putative colorectal CSC markers in HCT116 and HT29 cells treated with 50 or 100 μg/ml of OPCs. (The full-length western blot images are shown in supplementary Figs S2 and S3). (**E**) Expression of putative stemness-suppressive miRNAs, miR-34a (top) and miR-200c (bottom), in HCT116 and HT29 cells treated with different doses of OPCs (**F**) Left: Schematic of SDCSC-derived xenograft tumor pre-treated for 24 hours with OPCs. Right: Progressive SDCSC-derived xenograft tumor volume. *p < 0.05, **p < 0.01, ***p < 0.001 compared to control treatments.

Cancer stem cells play a pivotal role in various key oncogenic processes including tumor initiation, metastasis and chemoresistance^10,11^. In order to study the effect of OPCs on CRC stem cells, we generated spheroids from HCT116 and HT29 cells by culturing them in ultra-low attachment dishes under serum-free and stem cell-inducing conditions (Fig. 2A). As expected, we noted an upregulation of cancer stem cell markers LGR5 and CD44, as well as epithelial-to-mesenchymal transition marker ZEB1, in the spheroids (Fig. 2B). Hence, following confirmation for the enrichment of cancer stem-like cells in spheroids, we used these as a model system to further investigate the effects of OPCs on CRC stem-like cells. When we cultured spheroids in the presence of OPCs for five days, we observed a significant decrease in the number of spheroids formed (all treatments were P < 0.001 compared to respective control for both cell lines except for 50 µg/ml HT29 treatment) (Fig. 2C). Furthermore, OPCs downregulated several well-established large intestinal cancer stem cell markers such as CD133, CD44 and LGR5, indicating that OPCs particularly target cancer stem cells in CRC (Fig. 2D). Intriguingly, the expression of Notch1, a major regulator of self-renewal in CRC, and cleaved-Notch1, an active component of Notch intercellular domain, were also suppressed by OPCs in CRC cells (Fig. 2D).

To further investigate the self-renewal suppressive roles of OPCs, we assessed the expression of well-characterized stemness-suppressing miRNAs, miR-34a and miR-200c, following OPC treatment in both HCT116 and HT29 cells (Fig. 2E). MiR-34a is a well-recognized tumor suppressor that targets CRC stem cells by regulating Notch1 and CD44 expression^24^, while miR-200c plays a major inhibitory role in ZEB1 regulation^25,26^. We showed that 200 µg/ml treatment of OPCs resulted in upregulation of miR-34a and miR-200c in both cell lines (all p < 0.05) suggesting that OPCs may in part regulate self-renewal genes through epigenetic modulation. In addition, OPCs also suppressed the expression of oncogenic miRNAs, miR-21 and miR-27a, which are known to be involved in stemness regulation^27–29^ (Supplementary Fig. 1A).

Finally, to demonstrate that OPCs inhibit tumor initiation potential of CRC cells *in vivo*, we pretreated cancer stem-like cell-enriched spheroids with 50 and 100 μg/ml OPCs or DMSO (as vehicle) for 24 hours and injected these cells into the flanks of immune-deficient mice and monitored the growth of tumors for two weeks (Fig. 2F, left). Consistent with our *in vitro* findings, xenograft tumors derived from spheroids pre-treated with OPCs resulted in significantly smaller tumors compared to controls (both p < 0.001, OPC extract treatment groups vs. controls; Fig. 2F middle and right). Collectively, our data demonstrated that OPCs inhibit both cancer stem cell formation, as well as tumor growth with corresponding suppression of cancer stem cell markers.

### OPCs suppress growth of mouse and patient derived tumor organoids

**Figure 3:**
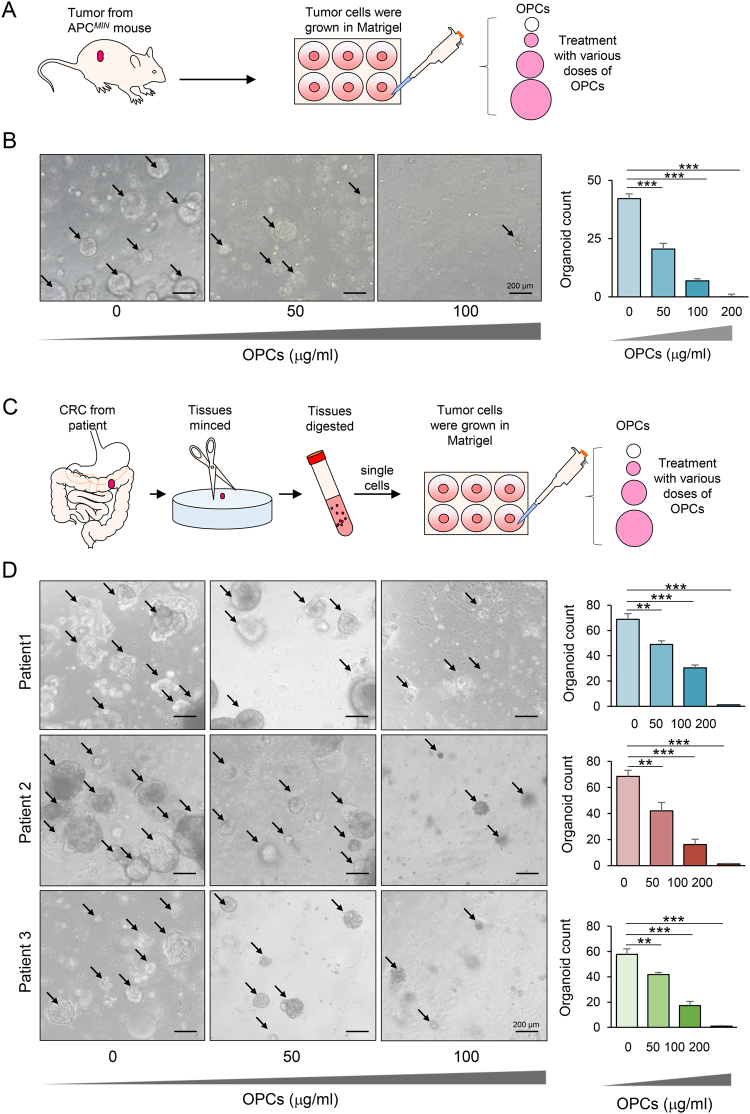
OPCs inhibit growth of APC^*MIN*^ mouse and patient-derived tumor organoids. (**A**) Schematic protocol of OPCs treatment on APC^*MIN*^ mouse derived organoids (**B**) Images showing APC^*MIN*^ mouse derived organoids treated with 0–100 µg/ml of OPCs (left). Bar graph showing decrease in organoid count with OPCs treatment (right) (**C**) Schematic protocol of OPCs treatment on patient-derived tumor organoids. (**D**) Images showing three independent patient-derived tumor organoids treated with 0–100 µg/ml of OPCs (left). Bar graph showing decrease in organoid count with OPCs treatment (right). **p < 0.01, ***p < 0.001 compared to control treatments.

To further validate that OPC enriched grape seed extract suppresses the formation of cancer stem cells and subsequently inhibits tumor growth, we generated tumor organoids from an *APC*^*Min*^ mouse, using a previously established method (Fig. 3A)^30^. The *in vitro* organoid model utilizes stem cell mediated formation of self-renewing tissues and is recognized as an important tool to accurately investigate various biological processes owing to its near physiological 3D architecture^20^. We treated the tumor organoids with various doses of OPCs and observed their growth. Consistent with our cell line data, OPCs suppressed the formation of mouse-tumor organoids (all p < 0.001 compared to the controls), as well as inhibited the overall organoid growth (Fig. 3B).

Subsequent to our demonstration for suppression of cancer stem-like cell growth by OPCs using spheroids derived from human cell lines and mouse tumors, we wanted to further confirm our findings using patient-derived tumor organoids. In order to achieve this, we harvested cancer cells from three independent CRC patients and generated colorectal tumor organoids, and assessed the efficacy of OPCs in these tumor organoids (Fig. 3C). Consistent with our findings in cell lines and mouse tumor organoids, OPCs consistently suppressed the formation as well as the growth of patient-derived tumor organoids (all p < 0.01 compared to controls; Fig. 3D). Considering that organoids are generated and expanded from stem cells, these data not only highlight that OPCs inhibit the growth of cancer stem cells, but successfully demonstrate that this effect is mediated through targeting of cancer stem cells and OPCs suppressed subsequent tumor formation.

### OPCs alter molecular profile of CRCs

**Figure 4:**
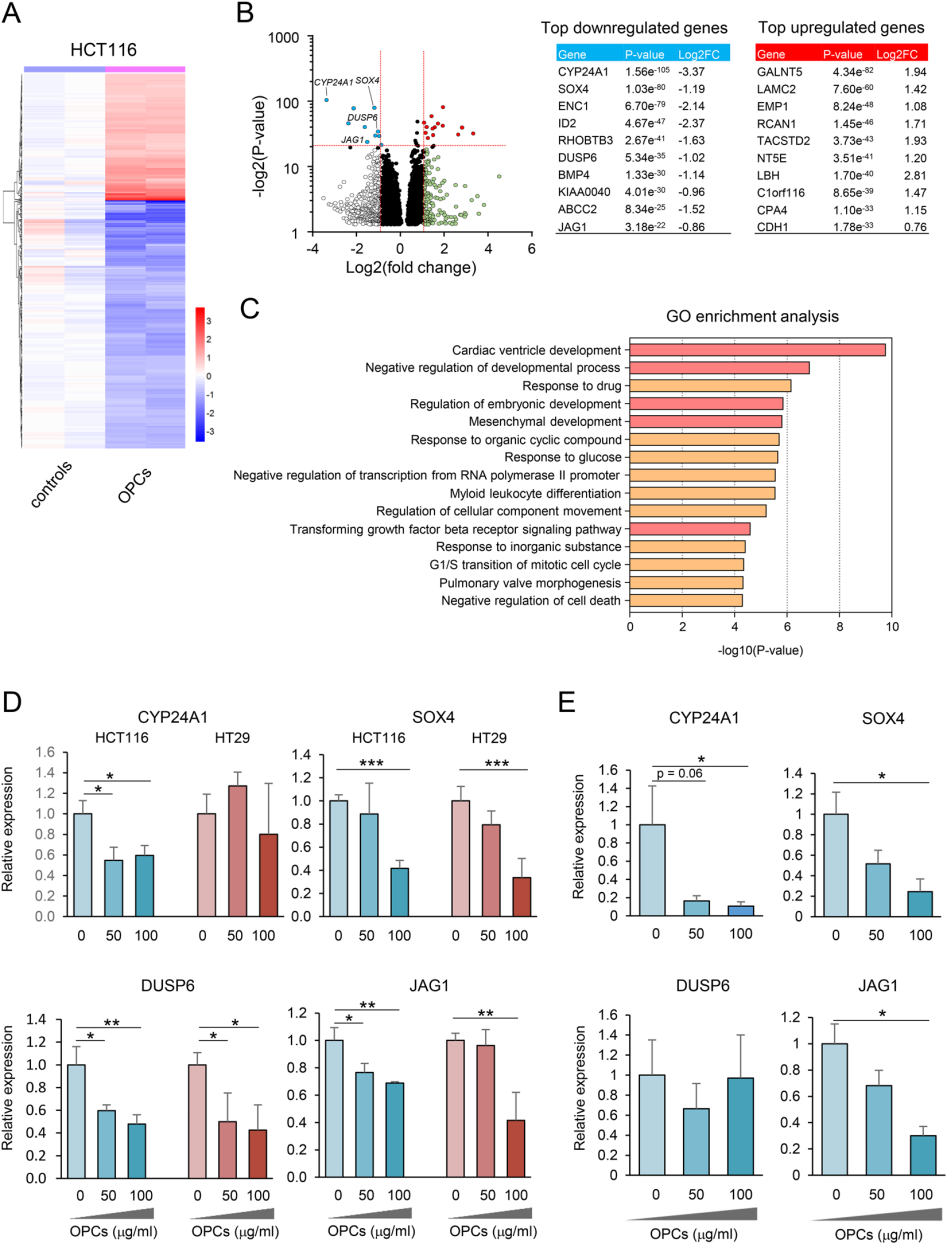
OPCs modulate developmental genes/pathways (**A**) Heat map of differentially expressed genes with or without OPCs treatment in HCT116 cell line. (**B**) Volcano plots of differentially expressed genes identified from RNA-sequecing. Most highly differentially downregulated genes are highlighted in blue and upregulated genes are highlighted in red and summarized in left tables. (**C**) List of pathways identified by Gene Ontology (GO) enrichment analysis. Developmental pathways are highlighted in pink (**D**) qPCR analysis of putative self-renewal associated genes in HCT116 and HT29 CRC cell lines treated with OPCs. (E) qPCR analysis of putative self-renewal associated genes in patient-derived organoids treated with OPCs. * p < 0.05, ** p < 0.01 ***p < 0.001.

To investigate the underlying molecular mechanisms of OPCs, we conducted RNA sequencing to profile gene expression alteration of HCT116 CRC cells following treatment of OPCs. We initially identified 3,551 differentially expressed genes of which 1,625 were upregulated and 1,926 were downregulated (Fig. 4A). Subsequently, using a criteria based on fold change and p-values (highest p-values with at least log2 fold change of 0.75), we identified several genes which were most differentially expressed (Fig. 4B). Intriguingly, several putative self-renewal associated genes were found in the top downregulated genes (CYP24A1, SOX4, DUSP6 and JAG1)^31–34^. Furthermore, Gene Ontology enrichment analysis revealed that multiple developmental/self-renewal pathways were altered by OPCs (Fig. 4C), suggesting that OPCs are likely to interfere with these pathways. To further confirm the inhibition of these self-renewal suppressing genes, we assessed the expression of key genes by qPCR. Consistent with our sequencing data, SOX4, DUSP6 and JAG1 were downregulated by OPCs in both HCT116 and HT29 cell lines (p < 0.05, control vs. 50 & 100 μg/ml OPC treatment for both cell lines, all three genes; Fig. 4D). We thereafter assessed the expression of these genes in patient-derived organoids. In line with our cell line data, OPC treatment inhibited the expression of CYP24A1, SOX4 and JAG1 (all p < 0.05; Fig. 4E). These data highlight the consistent suppression of stemness-associated putative oncogenes by OPCs in both CRC cell lines and tumor organoids.

**Figure 5:**
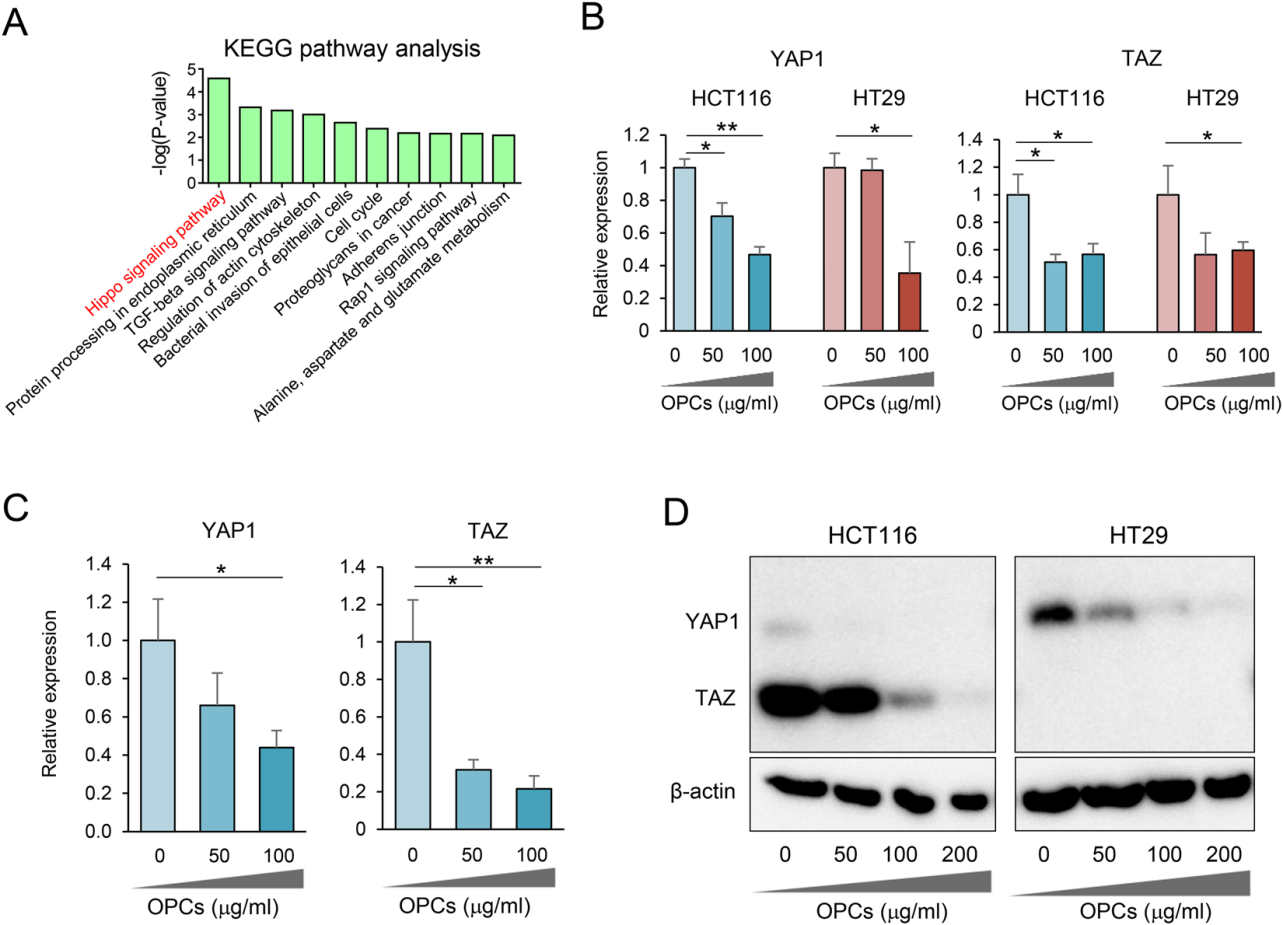
OPCs suppress Hippo pathway. (**A**) Top pathways identified by KEGG analysis ranked by p-values. (**B**) qPCR analysis of YAP and TAZ expression in HCT116 and HT29 cell lines treated with 0–100 µg/ml of OPCs. (**C**) qPCR analysis of YAP and TAZ in patient-derived tumor organoids treated with 0–100 µg/ml of OPCs. (**D**) Protein expression of YAP, TAZ with OPCs treatment in HCT116 and HT29 cell lines. (The full-length western blot images are shown in supplementary Fig. S4). *p < 0.05, **p < 0.01 ***p < 0.001.

Next, we analyzed our sequencing data using KEGG pathway analysis to identify possible signaling pathway(s) that OPCs may interact. Intriguingly, the pathway analysis revealed that OPCs modulated key pathways in cancer including, Hippo signaling pathway, TGF-β signaling pathway and cell cycle regulatory pathways (Fig. 5A). In particular, Hippo pathway has been implicated in reprogramming of non-stem tumor cells to cells with cancer stem cell attributes^35,36^. Therefore, we investigated whether OPCs influence the Hippo pathway. Initially we assessed the expression levels of YAP and TAZ, two major putative oncogenes in this pathway. Surprisingly, the expression of YAP and TAZ was downregulated by OPCs in both HCT116 and HT29 cell lines (p < 0.05, OPC treatment vs. controls; Fig. 5B). We thereafter showed that suppression of YAP and TAZ by OPCs were consistent in patient-derived tumor organoids (Fig. 5C). To ensure that these oncogenes are suppressed at post-transcriptional levels, we used western blot analysis to confirm that OPCs inhibited the protein expression of YAP and TAZ in both HCT116 and HT29 CRC cell lines (Fig. 5D). Collectively, these results demonstrated that OPCs inhibit Hippo pathway, which may in part explain the underlying mechanisms of OPCs to inhibit cancer stem cell formation and self-renewal capacity.

### OPCs inhibit tumor growth in a xenograft animal model

**Figure 6:**
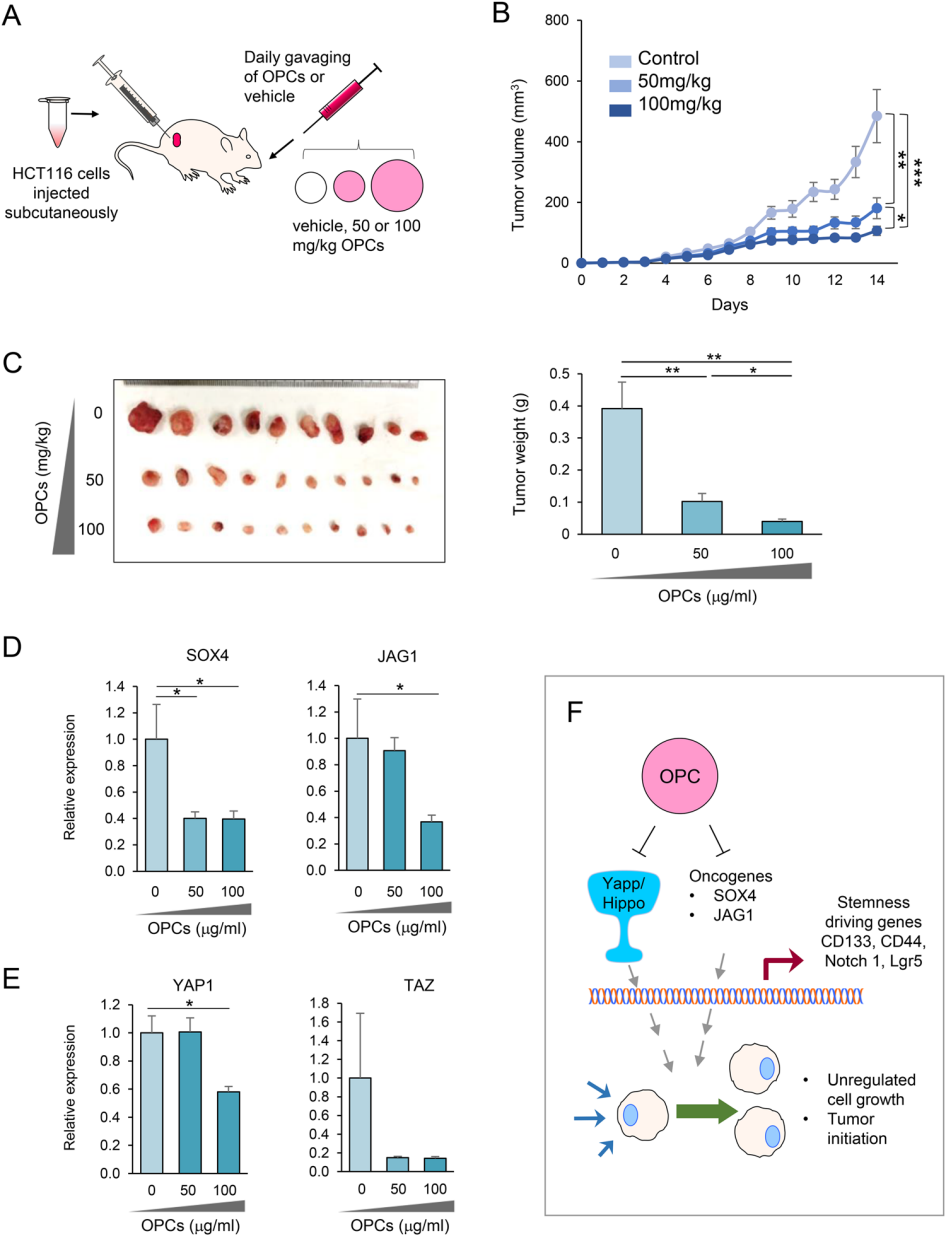
Oral intake of OPCs suppresses xenograft tumor growth **(A**) Schematic of OPCs gavaging of mice xenografted with HCT116 cells. (**B**) Progressive tumor volume in mice gavaged with OPCs. (**C**) Left: Xenograft tumor collected from sacrificed mice at the end of 14-day treatments. Right: Average tumor weight of resected tumors (**D**) qPCR analysis of SOX4 and JAG1 expression in xenograft tumors (**E**) qPCR analysis of YAP and TAZ expression in xenograft tumors (**F**) Model showing OPCs suppression of cancer cell growth by inhibiting Hippo pathway and onocogenes such as SOX4 and JAG1. *p < 0.05, **p < 0.01, ***p < 0.001.

In order to further substantiate the anti-tumorigenic properties of OPCs that were observed *in vitro*, we generated HCT116-xenograft tumors in athymic nude mice and treated them daily with 50 mg/kg or 100 mg/kg of OPC extract or vehicle through oral gavaging (Fig. 6A). We deliberately chose relatively low doses of OPCs to mimic physiologically-relevant doses. Based on the human equivalent dose estimate, daily consumption of 50 and 100 mg/kg of OPCs for a mouse yield approximately 284 and 568 mg/day for a person with an average 70 kg weight^37^. Both 50 and 100 mg/kg concentration of OPCs dissolved well in water. At day 6 of the treatment, 100 mg/kg OPCs treated group displayed lower tumor growth (p < 0.05) compared to the controls, while reduced growth was observed for 50 mg/kg OPCs treated group starting day 9 (p < 0.05). Both OPC treatment groups resulted in significant reduction in tumor growth compared to controls after two weeks of gavaging (Fig. 6B). Consistently, tumor weight of OPC treated animals was lower compared to controls (both p < 0.01) (Fig. 6C).

We finally assessed whether treatment with OPCs altered the expression of genes that we identified in our *in vitro* experiments, in the xenograft tumors as well. We assessed the expression of SOX4 and JAG1 as well as Hippo pathway associated oncogenes, YAP and TAZ. Analogous to our *in vitro* data, OPC treatment resulted in suppression of SOX4, JAG1 and YAP1 (all p < 0.05) (Fig. 6D,E). Collectively, our data highlight that oral intake of OPCs resulted in significant reduction in tumor growth with corresponding inhibition in self-renewal associated genes.

## Discussion

Unlike other cancer types, majority of sporadic CRCs are preventable through lifestyle and dietary modifications. Therefore, it is not surprising that traditional alternative medicines have been identified as possible chemopreventive agents in this malignancy. Although many botanical agents have been used to treat various diseases for centuries, only recently have we begun to understand their underlying chemopreventive mechanisms. Herein, we demonstrated the anti-tumorigenic properties of OPCs, from a purified grape seed extract, in a series of *in vitro*, *in vivo* and patient-derived organoid experimental models. We provide multiple layers of evidence supporting anti-cancer effects of OPCs through a significant attenuation of cancer stem cell formation in various model systems. Mechanistically, we profiled OPC-induced gene expression alterations by RNA-sequencing to confirm that OPCs predominantly inhibited developmental and self-renewal pathways and identified several novel target oncogenes targeted by OPCs. Furthermore, we validated inhibition of Hippo pathway by OPCs. Finally, we for the first time utilized patient-derived colorectal tumor organoids to confirm the anti-tumorigenic properties of OPCs observed in both *in vitro* and *in vivo* experiments.

Over the last several years, the organoid model has been recognized as an ideal *in vitro* tool for linking basic biology to clinical applications. The organoid model allows maintenance and expansion of cancer stem cells as well as growth of differentiated cells providing a more realistic *in vitro* setting to mimic tumor growth. Therefore, it is not surprising that the organoid model is recognized as a facile model to study various diseases such as cancer and is currently considered as an ideal model for drug screening compared to other conventional 2D cultures and *in vivo* mouse models^20,21^. While other assays such as colonosphere formation are useful for studying the biological function of cancer stem cells, these models only enrich for cancer stem-like cells and are unable to assess how these cancer stem-like cells initiate the formation and progression of a tumor^38^. In contrast, the organoid model facilitates self-organized structural architecture through expansion of stem cells^20^. Herein, we demonstrated that patient-derived tumor organoids is a useful tool to test the efficacy of botanicals such as OPCs. While there are obvious experimental limitations with the organoid model, such as these organoids are typically restricted in matrigel, this model provides relatively simple and effective means to test the efficacy of natural compounds. Collectively, this model may provide a unique approach for interrogating the ‘precision medicine’ potential of various chemopreventative compounds.

Although the efficacy of botanicals maybe deemed somewhat modest compared to classic chemotherapeutic drugs, the safety of these compounds as dietary supplements is well established. Accordingly, several clinical pilot studies are currently underway to test the efficacy of botanical agents in various cancers^2–4^. Accumulating evidence suggests that several botanicals including curcumin, green tea extracts and grape seed extracts inhibit the formation of cancer stem cells and tumor initiation^16–19^. Thence, there is growing enthusiasm to therapeutically target cancer stem cells using dietary agents, individually or through their use as adjunctive treatments together with modern chemotherapeutic drugs^15^. In particular, curcumin, one of the most extensively studied botanicals, has been shown to inhibit cancer stem cell formation in multiple cancer types including head and neck, colon, ovarian and pancreatic cancers^18,39,40^. Several studies have consistently shown that grape seed extract also inhibits the formation of cancer stem cells in breast and colon cancers^41,42^. OPCs is a subset of condensed, tannins extracted from grape seeds, purified specifically for oligomers. In addition to *in vitro* interrogations, we were able to demonstrate that OPCs inhibited patient-derived tumor organoid formation that more closely recapitulate the clinical scenario, further supporting that OPCs are likely to target cancer stem cells which drive organoid formation^30^. To our knowledge, this is the first study to utilize patient-derived tumor organoids to test the efficacy of such botanicals. Our study also highlights the potential for using such a model system for evaluating the efficacy of dietary compounds against human tumors.

Accumulating studies have revealed that ‘stemness’ in cancer cells is regulated by multiple inter-linked mechanisms^14^. Accordingly, our RNA-sequencing data indicate that OPCs interfere with multiple self-renewal associated pathways. We identified one such self-renewal associated pathways, Hippo pathway, which is inhibited by OPCs through suppression of transcriptional activators, YAP and TAZ proteins, that are frequently overexpressed in human malignancies^35^. Recently, YAP and TAZ were shown to be actively expressed in the cancer stem cell-fraction and were required for their expansion and reprograming of non-stem tumor cells into cancer stem cells^36,43^. We demonstrated that OPC-enriched grape seed extract inhibited the expression of YAP and TAZ, suggesting that these compounds suppress self-renewing capacity of cancer cells, in part by suppressing Hippo pathway. In addition, we have identified unique putative oncogenes targeted by OPCs. JAG1, a Notch ligand, and is known to act as an oncogene through activation of Notch signaling pathway in CRC^44^; while SOX4 is a transcription factor involved in regulation of embryonic development and frequently over expressed in various cancers^45^. Although we have focused primarily on self-renewal associated genes in the present study, our data showed that OPCs are involved in multiple pathways highlighting their therapeutic potential.

In conclusion, we have comprehensively demonstrated chemopreventive efficacy of OPCs using multiple *in vitro* and *in vivo* models. In particular, the successful utilization of patient-derived tumor organoids supports feasibility of natural compound based precision medicine. Mechanistically OPCs inhibited the formation of cancer stem cells through the suppression of multiple self-renewal-associated pathways including Hippo pathway. Considering that OPC is a cost-effective non-toxic natural compound, it may provide a safe and effective therapeutic approach to target CSCs.

## Methods

### Cell culture and materials

HCT116 and HT29 CRC cells were purchased from the American Type Culture Collection (Manassas, VA). The cells were grown in Iscove’s Modified Dulbecco’s Medium (IMDM; Gibco, Carlsbad, CA), supplemented with 10% fetal bovine serum, 1% penicillin and streptomycin and maintained at 37 °C in a humidified incubator at 5% CO_2_. Spheroids were generated from HCT116 and HT29 cells and enriched for cancer stem cells by culturing them in serum-free medium (DMEM/F12) supplemented with B27 (Gibco), N2 (Gibco) and 10 ng/ml human recombinant basic fibroblast growth factor (bFGF, Gibco) and 20 ng/ml epidermal growth factor (EGF, Sigma-Aldrich) in Costar^®^ ultra-low attachment flask (Sigma-Aldrich, St. Louis, MO). French grape seed extract VX1 OPC extract (EuroPharma USA, Green Bay, WI), enriched for only small molecular-weight components (monomers, dimers and trimers) and devoid of tetramers and higher molecular weight tannins, was used in this study. For treatments, OPCs were dissolved in DMSO and diluted to appropriate experimental concentrations in culture medium.

### Viability, cell cycle, apoptosis and colony formation assays

Cells were incubated with various concentrations of OPCs for 72 hours in 96-well plates and cell proliferation was measured using MTT assays as described previously^46^. Cell cycle analysis was performed using the Cell Cycle Assay Kit (MCH100106; Millipore, Billerica, MA) and apoptotic cell fraction was measured using the Annexin V and Dead Cell Assay Kit (MCH100105; Millipore) on Muse Cell Analyzer (Millipore) according to the manufacturer’s instructions. Colony formation assays were performed as described previously^47^. The number of colonies (>50 cells) were counted using GeneTools (Syngene, Cambridge, UK). All experiments were performed at least in triplicates.

### RNA isolation, cDNA preparation and qRT-PCR analysis

RNA was extracted from CRC cell lines and xenograft tumor tissues using the miRNeasy Mini Kit (Qiagen, Germantown, MD) following the manufacturer’s instructions. In brief, 3 mm cube xenograft tumor tissues were homogenized using TissueLyser II (Qiagen) with 5 mm stainless steel beads. RNA was isolated using Qiacube (Qiagen) and eluted in 50 µl of RNase-free water. Organoids were harvested in RLT lysis buffer and RNA was isolated with the RNeasy Mini Kit (Qiagen) using Qiacube and eluted in 50 µl of RNase-free water. Extracted RNA was used as a template for cDNA production using High Capacity cDNA Revers Transcription Kit (Thermo Fisher Scientific) according to manufacturer’s protocol. RT-qPCR was performed using SensiFAST SYBR mix (Bioline, London, UK) on Quant-Studio 7 PCR machine (Applied Biosystems). For specific primer sequences refer to Supplementary Table 1. All RT-qPCR target genes were calculated using ΔΔCt method normalized to β-actin. For miRNA expression analysis, we used the TaqMan real-time PCR assay kit (Applied Biosystems, Foster City, CA) and TaqMan microRNA Reverse Transcription Kit (Applied Biosystems) as described previously^48^. For all reactions, TaqMan Universal Master Mix (Applied Biosystems) was used and the analysis was carried out using Quant-Studio 7 (Applied Biosystems). All data were analyzed using ΔΔCt method and normalized to RNU6B.

### RNA sequencing and analysis

NGS library construction was performed using the TruSeq RNA Library Kit (Illumina) with up to 1 µg of total RNA input according to manufacturer’s protocol. The quality of individual libraries was assessed using the High Sensitivity DNA Kit (Agilent). Libraries were pooled together using a Pippin HT instrument (Sage Science). Efficiency of size selection was assessed using a High Sensitivity DNA Kit (Agilent). Pooled libraries were quantitated via qPCR using the KAPA Library Quantification Kit, Universal (KAPA Biosystems) prior to sequencing on an Illumina HighSeq. 2500 with single-end 75 base read lengths. For the analysis of RNA-seq, fastq files were trimmed using Flexbar to remove 3′ bases with quality score lower than 30 before alignment as described previously^49^. The trimmed reads were mapped to human genome version GRCH38 downloaded from GENCODE^50^ using HISAT2^51^ to generate alignment files in bam format. Samtools name-sorted bam files^52^ were processed using htseq-count to summarize gene level counts as described previously^53^. DESeq2 was used for differential gene expression analysis of RNA-Seq read counts^54^. Significantly differentially expressed genes based on FDR < 0.05 were uploaded to The Database for Annotation, Visualization and Integrated Discovery (DAVID) for functional annotation^55^ and Gene Ontology Consortium (http://www.geneontology.org/) for analysis of enriched biological processes. All sequencing data have been deposited to Gene Expression Omnibus under the accession code GSE109607.

### Western Immunoblotting

Western immunoblotting experiments were performed as described previously^56^. Cells were treated with various concentrations of OPC extract for 24 hours and lysed using 100 µl of 1 X SDS sample buffer containing β-mercaptoethanol. Primary antibodies used is listed in Supplementary Table 2. Anti-mouse or anti-rabbit secondary antibodies were from Santa Cruz Biotechnology (Dallas, TX). β-actin (Sigma-Aldrich) was used as the loading control.

### Sphere forming assay

HCT116 and HT29 cells were dissociated into single cells and seeded in a Costar ultra-low attachment 96-well plates (Sigma-Aldrich), in serum-free stem cell medium. Spheroids were treated with OPCs 24 hours after seeding. Spheres were counted using a light microscope (Olympus, Tokyo Japan) following 5-day incubation.

### Mouse and patient-derived organoids

Tumors were collected from *APC*^*Min*^ mouse and CRC patients enrolled at the Baylor University Medical Center, Dallas. Gastrointestinal tumor cells were cultured using a modified protocol described previously^30^. Briefly, following excision, tumors were maintained in a medium containing DMEM-F12 (Gibco) supplemented with 1% HEPES (Sigma-Aldrich), 1% L-glutamine (Gibco), 10% FBS (Gibco), 2% penicillin/streptomycin (Sigma-Aldrich) and 10 μM Y-27632 (R&D Systems). Tissues were minced and digested with collagenase solution (5 ml of above medium with 75 μl collagenase, 124 μg/ml dispase type II and 0.2% Primocen) for 30 min, and then filtered through a 70 μm filter (Corning). Cells were pelleted by centrifugation (200 g for 10 min) and suspended in Matrigel (BD Biosciences, Franklin Lake, NJ). 15 μl of the cell-Matrigel suspension was placed in the center of 24-well plate and polymerized. A 1:1 mixture of L-WRN conditioned medium and DMEM/F12 medium (Gibco) supplemented with 20% FBS (Gibco), 2mM L-glutamine (Gibco), 0.2% Primocen, 10 μM Y-27632 (R&D Systems), 10 μM SB431542 (R&D Systems) and 5% penicillin/streptomycin (Sigma-Aldrich) were added to the well and replaced every two days. For treatment of mouse and patient derived organoids, appropriate concentration of OPCs was added to the culture medium and cultured for 10 days. The animal protocol was approved by the Institutional Animal Care and Use Committee, Baylor Scott & White Research Institute, Dallas, Texas in accordance to the National Institute of Health Guide for the Care and Use of Laboratory Animals (8th Edition Institute for Laboratory Animal Research) and the human protocol was approved by Institutional Review Board, Baylor Scott & White Research Institute, Dallas, Texas. Written informed consent was obtained from all patients providing specimens and tissue samples were obtained in accordance to the Declaration of Helsinki.

### Animal experiments

Seven week-old male athymic nude mice (Harlan Laboratories, Houston, TX) were housed under controlled conditions of light and fed *ad libitum*. Spheroid-derived xenograft tumors were generated as follows: HCT116-spheroids were treated for 48 hours with OPC extract (50, 100 μg/ml) or control (DMSO). Thereafter, 1 × 10^6^ cells from HCT116-spheroids were suspended in Matrigel matrix (BD Biosciences) and subcutaneously injected into flanks of mice using 27-gauge needle (n = 4 per group). Tumor size was measured every other day by calipers for 24 days. For OPC gavaging experiment, xenograft tumors were generated by injecting 2 × 10^6^ HCT116 cells suspended in Matrigel subcutaneously. Mice were then gavaged daily with vehicle (water) or OPCs (50 mg/kg, 100 mg/kg body weight OPC dissolved in water) for 2 weeks (n = 10). Tumor volume was calculated using the following formula: 1/2(length × width × height). The animal protocol was approved by the Institutional Animal Care and Use Committee, Baylor Scott & White Research Institute, Dallas, Texas and conducted strictly in accordance to the National Institute of Health Guide for the Care and Use of Laboratory Animals (8th Edition Institute for Laboratory Animal Research).

### Statistical analysis

All analyses were performed using GraphPad Prism Ver. 6.0 (GraphPad Software Inc. San Diego, CA). All data were expressed as mean ± SEM with statistical significance indicated when p < 0.05. Statistical comparisons between control and treatment groups were determined using unpaired t test or one-way ANOVA with Tukey’s post-hoc tests.

## Electronic supplementary material

Supplementary Information
