# Supplementary material for: Oligomeric proanthocyanidins (OPCs) target cancer stem-like cells and suppress tumor organoid formation in colorectal cancer
Source: Sci Rep. 2018 Feb 20;8:3335. doi: 10.1038/s41598-018-21478-8 (PMC5820273; doi:10.1038/s41598-018-21478-8)

# Supplementary Information

# Oligomeric proanthocyanidins (OPCs) target cancer stem-like cells and suppress tumor organoid formation in colorectal cancer

Shusuke Toden, Preethi Ravindranathan, Jinghua Gu, Jacob Cardenas, Madelaine Yuchang and Ajay Goel

**Supplementary Table 1: Primer sequences**

| Primers | | Sequence |
|---|---|---|
| YAP1 | F | 5’- GAA CTC GGC TTC AGG TCC TC |
| | R | 5’- TCA TGG CAA AAC GAG GGT CA |
| TAZ | F | 5’- CAC CGT GTC CAA TCA CCA GTC |
| | R | 5’- TCC AAC GCA TCA ACT TCA GGT |
| CYP24A1 | F | 5’- GGT GGC GAG ACT CAG AAC G |
| | R | 5’- GTC GTG CTG TTT CTT GAG ACC |
| SOX4 | F | 5’- TTT CTC AGT GTG TGT GTT TAT TCC T |
| | R | 5’- TGG CAT CTG ATT ACA TTT ACA AGG |
| DUSP6 | F | 5’- ACC GAC ACA GTG GTG CTC TA |
| | R | 5’- AAC TTA CTG AAG CCA CCT TCC A |
| JAG1 | F | 5’- ACA CGG TCT CGG ATC AGG |
| | R | 5’- AGA AGT GGG AGC TCA AAG ACC |
| ACTB | F | 5’- AGA GCT ACG AGC TGC CTG AC |
| | R | 5’- AGC ACT GTG TTG GCG TAC AG |

**Supplementary Table 2: Antibody list**

| Antibody | manufacturer | |
|---|---|---|
| LGR5 | Abcam | ab75850 |
| CD44 | Abcam | ab119863 |
| ZEB1 | Santa Cruz | 10570 |
| CD133 | Millipore | 4399 |
| Notch1 | Cell Signaling | 4380P |
| Cleaved Notch1 | Cell Signaling | 4147P |
| YAP/TAZ | Cell Signaling | 8418P |
| EZH2 | Cell Signaling | 5246 |
| SUZ12 | Cell Signaling | 3737 |
| BMI1 | Cell Signaling | 6964P |
| Beta actin | SIGMA | A5441 |

**Figure legend**

Supplementary Figure 1: OPCs suppresses oncogenic miRNAs. A) The expression of putative oncogenic miRNAs with or without OPCs treatment. *P< 0.05

Supplementary Figure 2: A) The full length Western blotting images for the cropped images shown in Fig. 2B and Fig.D

Supplementary Figure 3: A) The full length Western blotting images for the cropped images shown in Fig. 2D.

Supplementary Figure 4: A) The full length Western blotting images for the cropped images shown in Fig. 5D.

**Supplementary Figure 1**

**A**

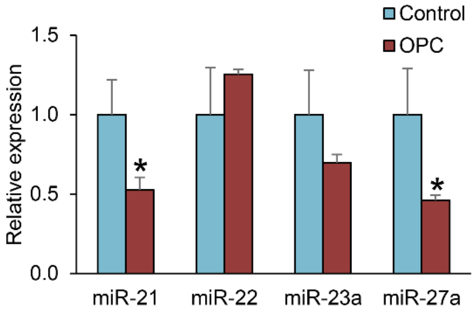

Supplementary Figure 2

A

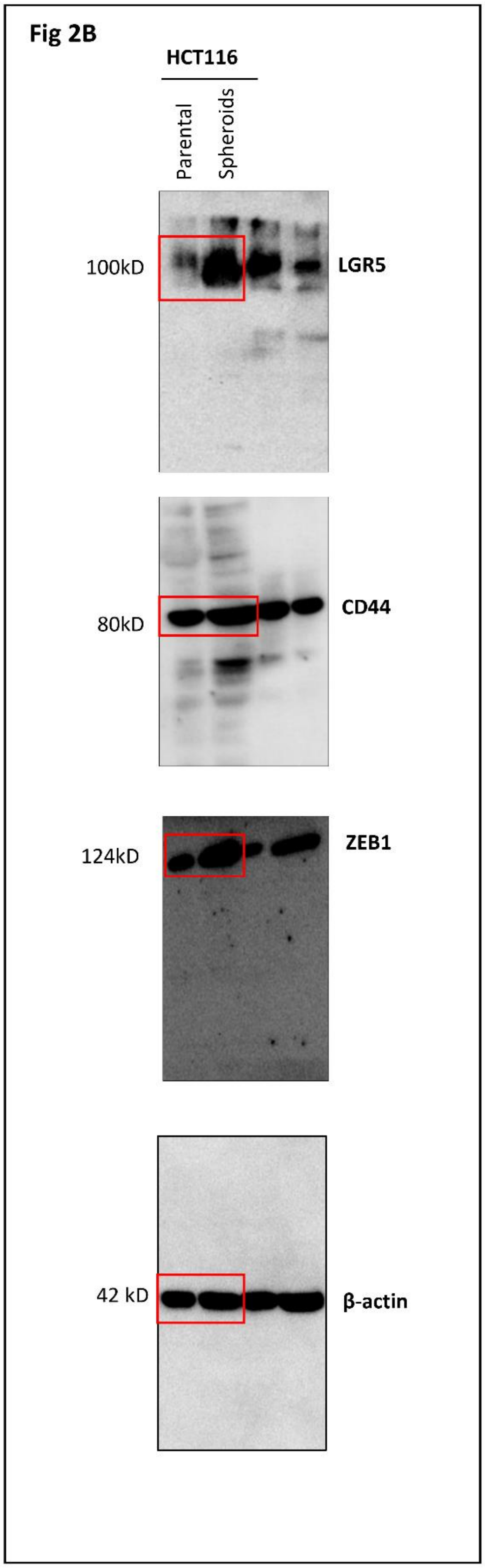


B

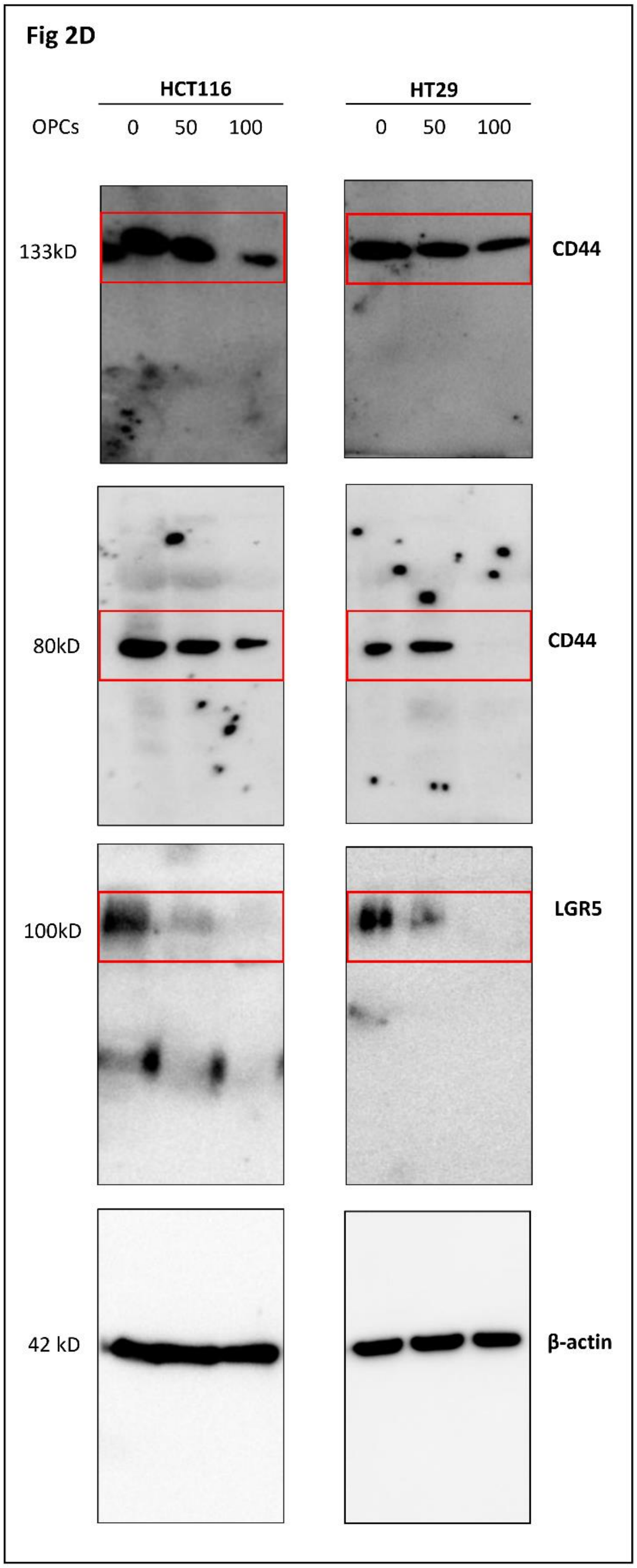

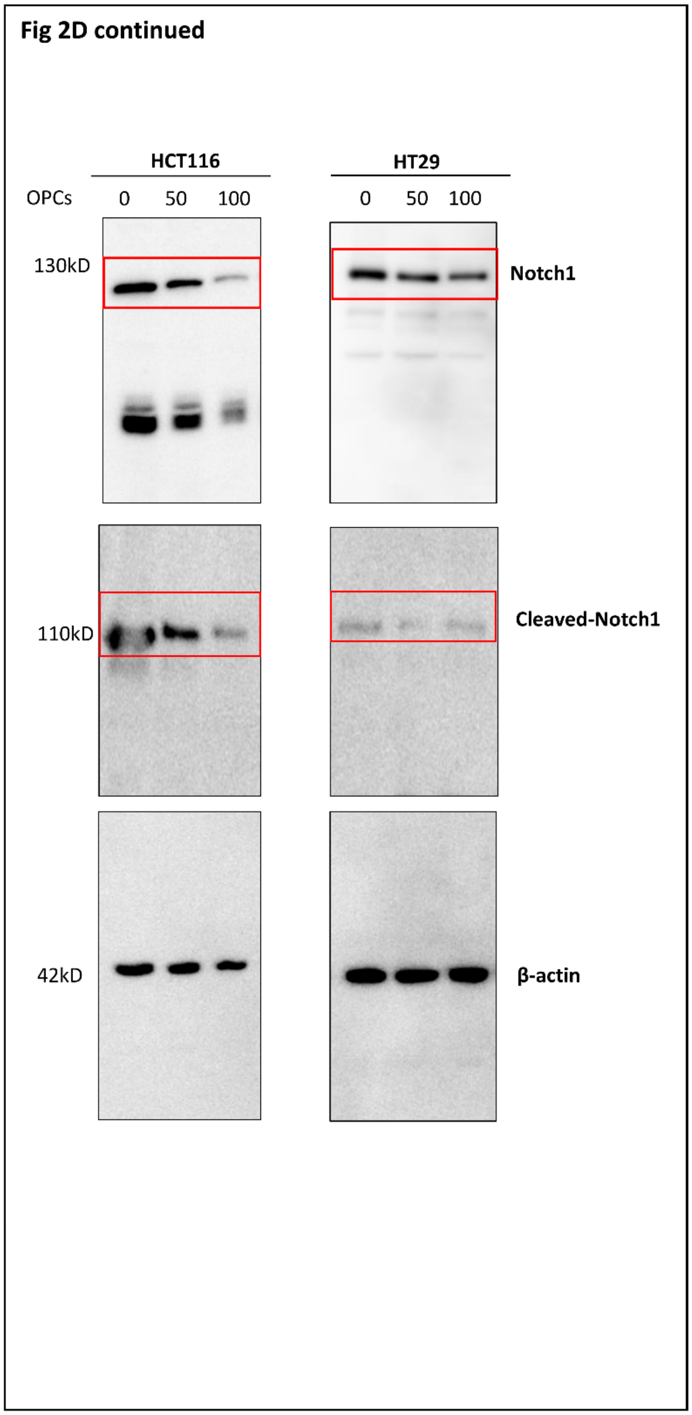
Fig 2D continued
HCT116
HT29
OPCs
0
50
100
0
50
100
130kD
Notch1
110kD
Cleaved-Notch1
42kD
β-actin

**Supplementary Figure 4**

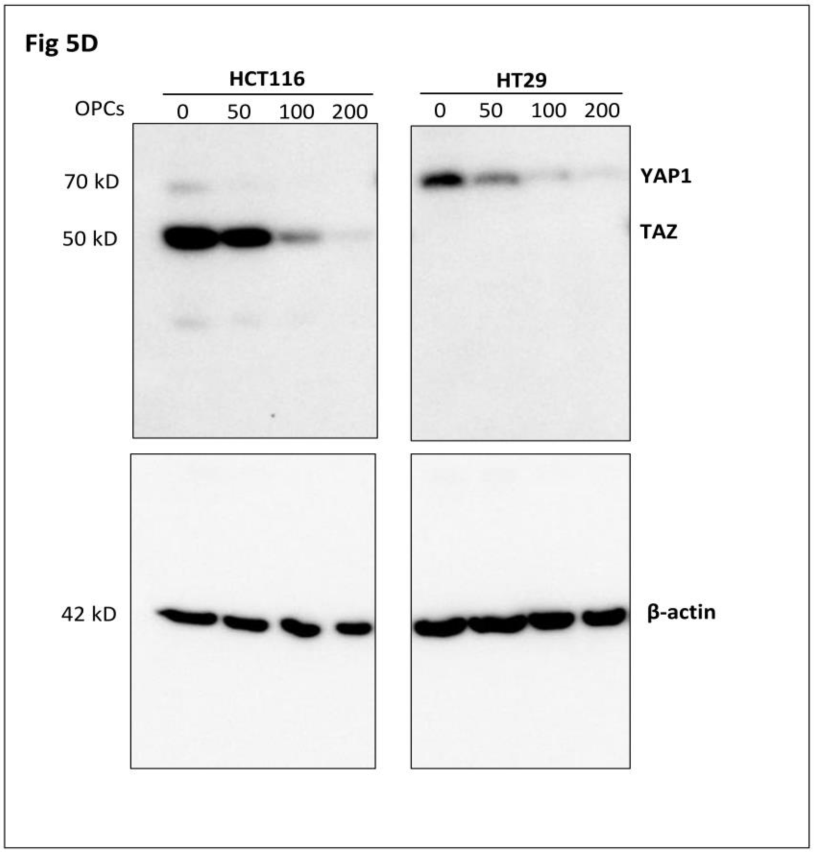

Supplement: Supplementary file 1 — Supplementary Information [file 41598_2018_21478_MOESM1_ESM.pdf]
